# Clinical Insights Into Linear Enamel Hypoplasia: A Case Report

**DOI:** 10.7759/cureus.116284

**Published:** 2026-09-15

**Authors:** Ramachandra Reddy Gowda Venkatesha, Karthik Rajaram Mohan

**Affiliations:** 1 Oral Medicine and Radiology, Vinayaka Mission's Sankarachariyar Dental College, Vinayaka Mission's Research Foundation (Deemed to be University), Salem, IND

**Keywords:** dental enamel hypoplasia, mandibular tooth, molar incisor hypomineralization, tooth age, tooth cervix

## Abstract

Linear enamel hypoplasia (LEH) is a failure or aberration of enamel to form correctly, resulting in bands of decreased enamel on the tooth surface. Many reasons, including genetics, malnutrition, and childhood diseases, can cause LEH, which solely affects the neonatal line. LEH is a kind of environmental hypoplasia with many symmetrical and ring-like abnormalities. Amelogenesis is a one-time procedure, and any abnormality might result in permanent enamel abnormalities. Enamel, unlike other human tissues, cannot be restored. The consequences of enamel hypoplasia vary based on illness severity, etiological agent interaction, and patient age upon presentation. The article provides information on the etiology, epidemiology, clinical features, and differential diagnosis of LEH.

## Introduction

Linear enamel hypoplasia (LEH) is identified in populations due to amelogenesis disruption as a sign of stress. Stress in the form of trauma, diet, viral illnesses, and environmental variations can alter enamel matrix production. Stresses in any form leave persistent physical traces on the teeth. Molar incisor hypomineralization (MIH) is a type of enamel defect that is qualitative and caused by decreased mineral density. In contrast, enamel hypoplasia is a quantitative enamel defect resulting from decreased enamel matrix secretion. However, in the most severe cases of MIH, the hypomineralized enamel might fracture, resulting in tissue thickness loss following tooth eruption. Enamel hypoplasia is characterized by a loss in enamel thickness that occurs before tooth eruption and, if associated with a systemic insult, affects all teeth growing at the time. At the transition to normal enamel, hypoplastic enamel lesions have regular margins but tend to fracture under occlusal forces, which are unevenly rough and appear in chalky-white or brownish-discolored areas [1]. Enamel hypoplasia occurs when enamel development is disrupted. The teeth most typically implicated are the central incisors, lateral incisors, canines, and molars; enamel production occurs within the first year following birth [2].

## Case presentation

A 16-year-old female reported to our department for a routine dental checkup. She did not have any harmful habits from chewing or smoking tobacco. Her past medical history was non-contributory. She had no history of any drug intake for fever or illness. A general examination revealed her vitals were stable. The intraoral examination showed the presence of a linear furrow-like defect on the labial surface of the maxillary anterior teeth and only the maxillary first molars (Figure 1).

**Figure 1:**
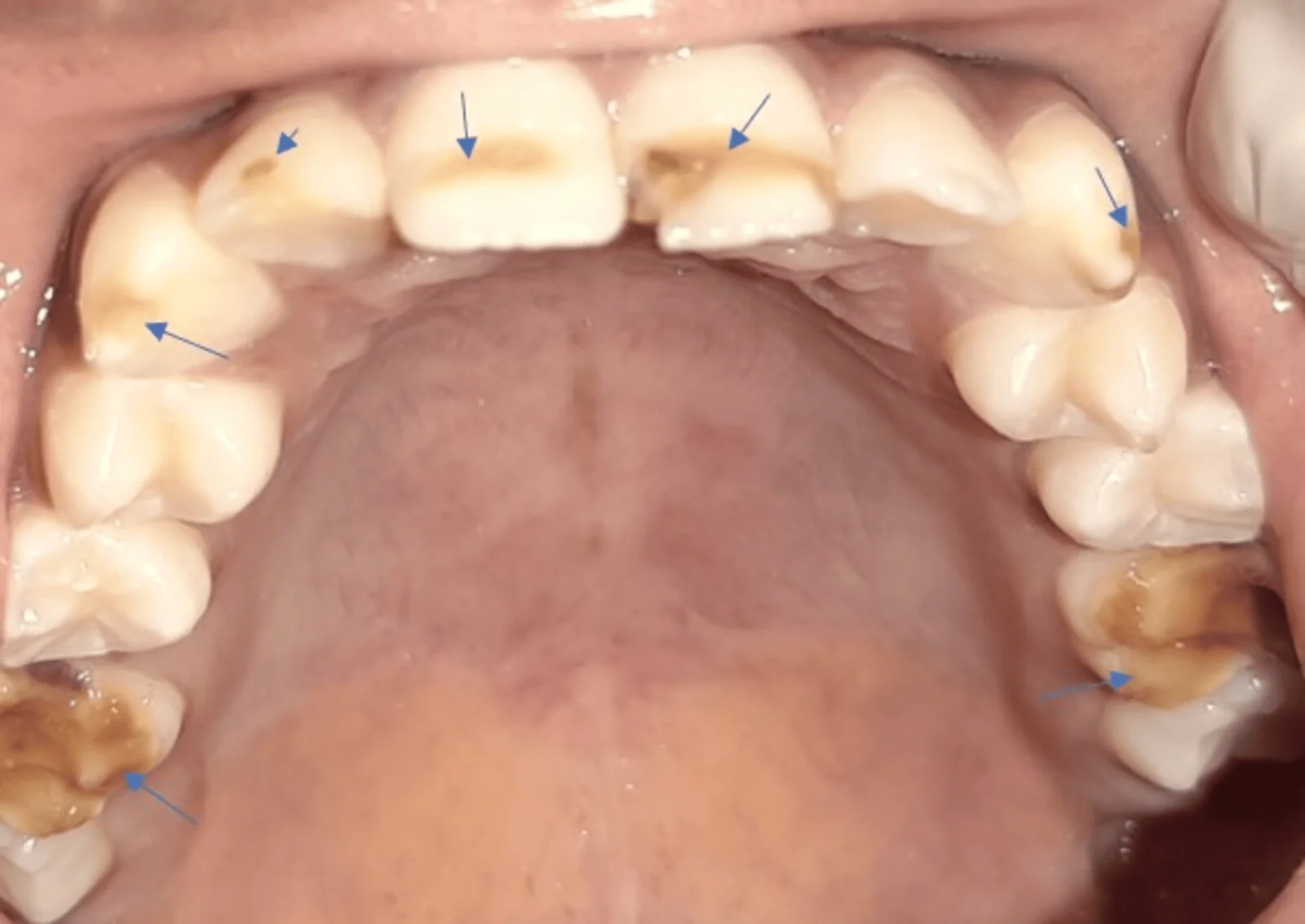
An intraoral examination showed a linear band-like hypoplastic defect on the middle-third of the enamel crown in the maxillary anterior teeth and on the occlusal surface of the maxillary first molars (blue arrows).

On further closer examination, a linear hypoplastic enamel defect was observed on the labial surfaces of the mandibular central, lateral, and incisal surfaces of the mandibular canine and first molars. The mandibular premolars and second molars are spared of any such hypoplastic enamel defects (Figure 2).

**Figure 2:**
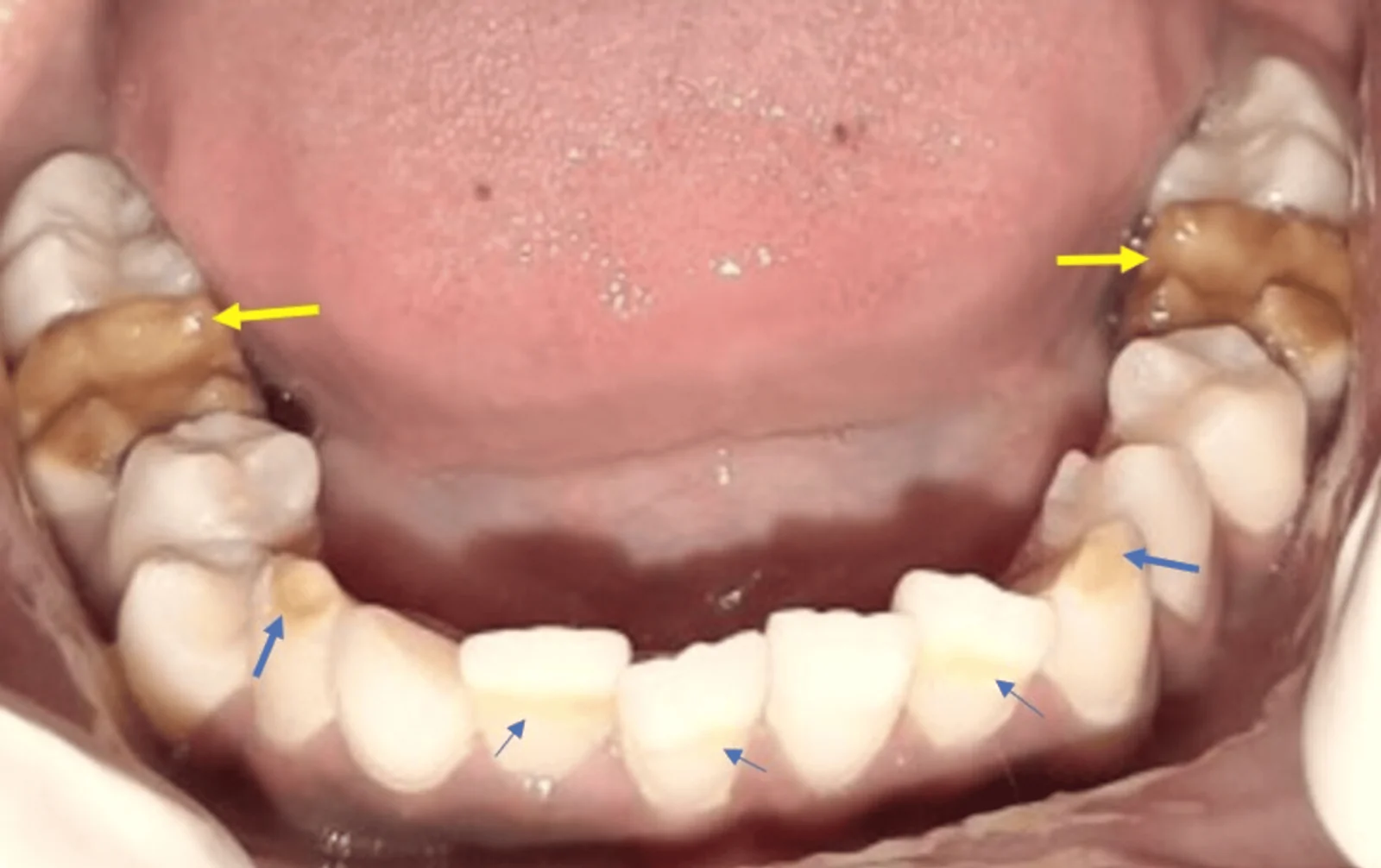
An intraoral examination revealed a furrow-like or band-like defect on the labial surface of the mandibular anterior teeth (blue arrows) and the mandibular first molar (yellow arrows).

The differential diagnosis for LEH includes MIH, amelogenesis imperfecta, Blaschko linear enamel defects, and odontoclasia. It is important to note that odontoclasia, a linear form of enamel caries in children that arises along the neonatal line, is not just a clinical condition but a social issue. It is commonly seen among impoverished children, highlighting the need for accessible dental care for all. The hypoplastic enamel defect in amelogenesis imperfecta occurs in all the teeth and occurs among families. Blaschko linear enamel defects run as vertical grooves extending along the middle third of the crown of the maxillary central incisors. They occur as a clinical marker in focal dermal hypoplasia, a genetic skin disease. The clinical significance of this case report lies in its description of a case of LEH resulting from stress induced by nutritional deficiency during the chronological eruption of the teeth between the ages of 9 and 11. Therefore, beyond this time of stress, the sequential erupted maxillary and mandibular premolars were not affected.

## Discussion

Definition

LEH refers to a visible alteration of the tooth crown surface, which may be observed under a microscope or with the naked eye. It appears as bands of different widths and depths that perpendicularly run around the tooth crown to its long axis [1].

Etiology

Enamel hypoplasia is primarily attributed to childhood illnesses and accidents. Hence, enamel hypoplasia can be regarded as a comprehensive marker of malnutrition and disease in elderly people [1]. The process of dental calcification is not independent but rather closely connected to the tooth's eruption and the development of the surrounding tissues [2].

The cause of enamel hypoplasia is linked to substances that disrupt the functioning of ameloblasts, which are cells responsible for generating enamel. This interference can lead to a decrease or complete halt in their activity. The deficiencies that can occur include protein deficiencies (such as amelogenin, enamelin, and tuftelin), vitamin deficiencies (A, C, K, and D), deficiencies of calcium, phosphorus, magnesium, or fluorine, diseases of the pituitary such as dwarfism, parathyroid disorders such as hypoparathyroidism, hypothyroidism, and Addison's disease. Bacterial infections such as tuberculosis and tetanus, viral infections such as rubella, measles, and herpes zoster, and spirochaetal infections such as syphilis disrupt the functioning of the ameloblasts [2].

Dental enamel hypoplasia refers to regions of reduced thickness in the enamel layer. These regions form when there is a disruption in the process of ameloblast deposition on the growing crowns of permanent and primary teeth. The production of enamel matrix is hindered due to increased levels of cortisol, which impede protein synthesis as a consequence of a stress episode. Numerous causes have been linked to their occurrence [3]. The distribution of abnormalities is very similar in terms of their position on the tooth crown; even though the chronology of enamel hypoplasia varies among teeth, the frequency of such LEH peaks near the crown's midway. The enamel prisms at this position on the crown are virtually perpendicular to the surface. This orientation may make it simpler to identify changes in rod lengths [3]. It was found that the rate of crown formation will rise at this period. The tooth was more vulnerable to interruption of enamel formation due to the rapid rate of enamel deposition during tooth development in these areas. Hypoplasia was characterized as circumferential bands, lines, or low pits with diminished enamel thickness [3].

Enamel hypoplasia is when the enamel becomes thinner due to the complete or partial suppression of ameloblast function during the secretory phase of enamel production. It can be classified into pits, lines, and grooves. The latter two are specifically known as LEH [4].

Methods of assessment of LEH

The various methods of assessment of LEH are presented in Table 1.

**Table 1:** Various methods to determine linear enamel hypoplasia (LEH). The table was modified and created with permission from the authors [3-11].

Author	Year	Method	Method of Calculation	Advantage	Disadvantages
Massler M and Schour I [3]	1941	Chart method	Age at LEH formation ¼ = - [1/velocity x distance of LEH from CEJ] + age at crown completion	A significant number of bioarchaeologists use them. Minimal computation tools	Charts do not possess separate surveys for females and males.
Ensor BE and Irish JD [4]	1995	Tooth hypoplastic area (THA) method	Acute hypoplasia or a one-stress event is given a value of 0.10. The crown disturbance percentage for chronic hypoplasia is calculated by dividing the defect's width by two-thirds of the estimated crown height. For example, if 70% of the crown is affected, the value assigned is 0.7%; if 50% of the crown surface is affected, it is 0.5%. The sum of these values is termed total hypoplastic area (THA).	Canines are commonly selected for analysis as they tend to have higher levels of hypoplasia and are more usually retained.	It is not possible to evaluate third molars. Assessing tooth loss caused by attrition is not possible. The majority of archaeological remains typically lack complete sets of teeth. The hypoplastic area approach may introduce a bias towards higher THA scores in persons with solely anterior polar teeth, as it relies on a percentage of crown surface enamel.
Reid DJ and Dean MC [5]	2000	Exponential regression equation Reid and Dean	Upper Central I 1= 5e^-0.146x^ Upper Lateral I 2 Age = 5.1e-^0.106x^ Upper Canine C1 Age = 5.3e ^-0.112x^ Lower Central I1 Age = 3.8e ^-0.135x^ Lower Lateral I2 Age = 4.2e ^-0.144x^ Lower Canine C1 Age = 6.2e -^0.141x^ ^a^Age in years (multiply by 365 for age in days). ^e ^is the mathematical constant. ^x^ is the distance of the defect from the CEJ in mm as a proportion of the crown height available. S is the Standard Error of the Regression.	Included central and lateral incisors and canines.	Not included molars for estimating linear enamel hypoplasia (LEH).
Reid DJ and Dean MC [6]	2006	Reid and Dean's method	X= d/(l/l-m) x 10 "x" is the distance of the defect from the CEJ along a standardized 10 mm tooth (d), the observed distance of the start of the defect from the CEJ in mm is divided by the length of the tooth available (i.e., length of the surface profile) in mm (l), which itself is divided by the percentage of the tooth that is missing (1-m) The crown is divided into deciles of crown height, and then the number of days required for each decile to develop is calculated.	The time required for the creation of the Cusp is taken into account. The genesis of LEH (lingual enamel hypoplasia) in molars was included. The decile approach incorporates latent cuspal. Factors such as enamel composition, the time of crown commence, and variations in crown height.	Enamel abnormalities that occur before the age of one are nearly indistinguishable on the surface of the tooth crown.
Guatelli-Steinberg D, Reid DJ (2008) [7]	2008	Micro polynomial method	The Micro Polynomial technique identifies flaws as depressions in the enamel surface that differ negatively by more than one standard deviation from a fitted sixth-order polynomial along the form. The observed features extend beyond the usual width of a single perikymata for the specific third of the tooth's crown in which they appear.	It relies on the depth of the enamel surface.	The accumulation of calculus on the surface of the enamel might result in enduring positive residuals. The steep angle at which these growth planes of enamel occur in the upper part of the tooth causes a greater distance between the ridges, resulting in enamel hypoplasia defects that are less deep and less well characterized.
Hasset [8]	2012	Micrometric method	Perikymata that are significantly deviated from a moving or local z-score by at least one standard deviation is amplified. Defects are documented on a perikymata-by-perikymata basis. The deviation of perikymata spacing from the moving average is calculated using a modified or local z-score. Potential differences are determined by measuring the consecutive lengths between two successive perikymata that deviate by more than one standard deviation from nearby neighboring values.		It requires a scanning electron microscope. It cannot be used when perikymata is not visible.
Gamble JA and Milne SB [9]	2016	Olympus LEXT 3D Laser Measuring Microscope OLS4000	The device is equipped with four objective lenses (5×, 10×, 20×, 50×), each of which may be digitally zoomed up to 8×. The magnification ranges from 108 to 864 times, with a field of view ranging from 2560 to 320 μm and a working distance of 20 mm.	It can be applied directly to the natural tooth or the dental model. Therefore, it is a non-invasive technique that does not necessitate lengthy sample preparation. Additionally, it generates images that directly measure the distance to the surface of the crown.	Expensive; it requires sophisticated software for analysis.
Cares Henriques A and Oxenham MF [10]	2019	Henriques and Oxenham method	a*e (b*x) a and b = constant coefficients for each type of tooth, e is the Euler's number.	This method is appropriate for deteriorated archaeological samples that do not have evident perikymata. It enables the unbiased identification and quantitative analysis of the sequence and length of defects. It also minimizes the chance of subjective judgment and enhances the precision and reproducibility of findings.	
Sensofar Plu Neox confocal microscope [11]	2024	Microscopic analysis	The vertical axis of each tooth crown (y-axis), from the CEJ to the incisal edge.	Resolution of 1.6 μm.	Linear enamel hypoplasia (LEH) measures are affected by the presence of depressions caused by pits, elevations, or fissures resulting from concretions or other materials attached to the enamel surface.

LEH, a dental anomaly associated with nutritional or other stress factors, was initially discovered in a Neolithic mammal in Hungary [12].

When the central region of the crown is impacted by hypoplasia, there is a noticeable alteration in the ratios of carbon and oxygen isotopes. This outcome is predictable since the development of LEH is connected to disruptions linked to the absorption of food, which is likely to be indicated by changes in the concentration of stable isotopes derived from the diet. LEH is caused by the physiological stress associated with the challenges encountered during the weaning stage [13].

Epidemiology

The prevalence of enamel hypoplasia was 12.8% [13]. The young individuals from Jabuticabeira II likely encountered frequent episodes of stress, potentially caused by infectious, diarrheal, and parasitic disorders. This stress was likely linked to introducing a "high-risk" diet, which may have included contaminated marine food in their supplementary and adult diets. LEH observed in adults indicates that these individuals have successfully overcome challenges experienced in early life. However, the presence of LEH in adolescents suggests a connection with an unidentified cause of death. In Jabuticabeira II, the individuals who managed to survive would have experienced frequent periods of illness (such as anemia, infections, and general stress), most likely caused by the high number of parasites in the environment and poor sanitation. Malnutrition, although possible, was probably not the leading cause of their health problems [14].

A comprehensive analysis was conducted on a combined total of 25 Italian cemetery remains and 139 migrants to assess the occurrence and intensity of LEH. This study has demonstrated that migrants have a higher frequency and greater severity of stress markers compared to the Italian group. Furthermore, these brain lesions are physical manifestations of the physiological disturbances they have endured. In reaction to stressors that affect the body's systems throughout growth, skeletal remains can show bone lesions, including cortical osteitis (CO), periosteal hyperostosis (PH), and LEH. The migrants' early life was characterized by poor living conditions and poverty, indirectly caused by social and economic inequalities. These conditions led to preventable biological harm, such as malnutrition, dietary insufficiencies, and infections. These bone lesions are a clear manifestation of the structural violence resulting from these circumstances [15].

LEH manifests as a dark-colored band that runs parallel to the incremental lines of enamel. This band is typically found between the neonatal line and the gingival margin in the incisors [15].

The high frequency and severity of adverse childhood experiences (ACEs) indicate a range of stressful events in a child's life [15]. The age range of faults and peaks in LEH production, as determined, was more remarkable for the maxillary teeth than for the mandibular teeth [15]. The prevalence of maxillary dentition was 27.6% for central incisors, whereas it was 35.4% for lateral incisors in mandibular dentition. The highest incidence of LEH defects was observed in young individuals (54.3%), followed by middle adults (50.0%), and the lowest frequency was recorded in old adults (40.0%) [15]. LEH of the deciduous maxillary incisors was observed in 73.1% of 104 Guatemalan children who were recuperating from their third stage of development [16]; 42.9% of the 150 youngsters undergoing treatment had second-degree malnutrition. The prevalence rates for these children from Guatemala are considerably higher compared to those reported for children from other regions of Guatemala. The correlation between an event that happened during the first month of life (linear hypoplasia) and the later occurrence of malnutrition (kwashiorkor and marasmus) may have epidemiological importance in identifying specific groups of children who would greatly benefit from food supplementation programs [16].

Differential diagnosis

The differential diagnosis for LEH includes MIH and Blaschko linear enamel defects. The teeth affected by MIH exhibit clearly defined boundaries of hypomineralized enamel, distinct from the opaque areas seen in fluorosis [17]. They were spread out over a wide area. Fluorosed enamel is more resistant to tooth decay than enamel afflicted by caries-prone MIH [18].

MIH is a distinct type of dental enamel defect (DDE) caused by systemic sickness and is defined by hypomineralization. This hypomineralization is observed as translucency in the enamel. Systemic factors often result in environmental enamel hypoplasia/hypomineralization, most typically observed in the first permanent molars and incisors. Enamel hypoplasia primarily affects the teeth that develop during the first year after birth, as indicated by clinical research. The incisors and primary permanent molars are the teeth that are most commonly affected. Therefore, this disease is referred to as MIH. When observed in a clinical setting, these abnormalities with less mineralization often deteriorate due to the pressure exerted during chewing. They appear as areas with a combination of underdevelopment and reduced mineral content. The premolars and the second and third molars are rarely impacted since their development commences at age three or later [18]. The vertical grooves on the teeth, known as Blaschko linear enamel defects, indicate the migration and growth of epidermal cells during embryonic development. These grooves are highly characteristic of focal dermal hypoplasia (FDH) [19].

Treatment

Remineralizing agent casein phosphopeptide-amorphous calcium phosphate (CPP-ACP) (Recaldent, GC Tooth Mousse, GC Corporation, Sharjah, United Arab Emirates) aids in the remineralization of hypoplastic areas of enamel in LEH. Composite restoration helps recontour the lost surface of the enamel defect caused by LEH [20].

## Conclusions

LEH must not be overlooked during a dental examination. It can cause problems with esthetics, tooth sensitivity, malocclusion, and an increased risk of dental caries. In its mildest form, LEH is often misdiagnosed as decay, as most teeth are prone to decay. Early recognition of LEH is critical because it primarily concerns aesthetics. Dentists have the chance to prevent these abnormalities through anticipatory guidance. A precise diagnosis is required to differentiate other illnesses and develop a conservative treatment approach if LEH is already present. Early diagnosis, treatment planning, and prognosis of LEH are crucial for preventing its complications from restoring esthetics and increasing masticatory efficiency.
